# Decision Aid for Colectomy in Recurrent Diverticulitis: Development and Usability Study

**DOI:** 10.2196/59952

**Published:** 2024-09-03

**Authors:** Alexander T Hawkins, Andrea Fa, Samuel A Younan, Srinivas Joga Ivatury, Kemberlee Bonnet, David Schlundt, Elisa J Gordon, Kerri L Cavanaugh

**Affiliations:** 1 Division of General Surgery, Section of Colon & Rectal Surgery Vanderbilt University Medical Center Nashville, TN United States; 2 Division of Colon and Rectal Surgery, Dell Medical School University of Texas at Austin Austin, TX United States; 3 Department of Psychology Vanderbilt University Nashville, TN United States; 4 Department of Surgery and Center for Biomedical Ethics and Society Vanderbilt University Medical Center Nashville, TN United States; 5 Division of Nephrology and Hypertension, Department of Medicine Vanderbilt University Medical Center Nashville, TN United States; 6 Vanderbilt Center for Effective Health Communication Vanderbilt University Medical Center Nashville, TN United States

**Keywords:** design sprint, diverticulitis, decision aid, shared decision-making, colectomy, diverticulitis, decision-making, diverticular diseases, gastrointestinal diagnosis, American, America, tools, tool, effectiveness, surgeon, patients, patient, communication, synopsis

## Abstract

**Background:**

Diverticular disease is a common gastrointestinal diagnosis with over 2.7 million clinic visits yearly. National guidelines from the American Society of Colon and Rectal Surgeons state that “the decision to recommend elective sigmoid colectomy after recovery from uncomplicated acute diverticulitis should be individualized.” However, tools to individualize this decision are lacking.

**Objective:**

This study aimed to develop an online educational decision aid (DA) to facilitate effective surgeon and patient communication about treatment options for recurrent left-sided diverticulitis.

**Methods:**

We used a modified design sprint methodology to create a prototype DA. We engaged a multidisciplinary team and adapted elements from the Ottawa Personal Decision Guide. We then iteratively refined the prototype by conducting a mixed methods assessment of content and usability testing, involving cognitive interviews with patients and surgeons. The findings informed the refinement of the DA. Further testing included an in-clinic feasibility review.

**Results:**

Over a 4-day in-person rapid design sprint, including patients, surgeons, and health communication experts, we developed a prototype of a diverticulitis DA, comprising an interactive website and handout with 3 discrete sections. The first section contains education about diverticulitis and treatment options. The second section clarifies the potential risks and benefits of both clinical treatment options (medical management vs colectomy). The third section invites patients to participate in a value clarification exercise. After navigating the DA, the patient prints a synopsis that they bring to their clinic appointment, which serves as a guide for shared decision-making.

**Conclusions:**

Design sprint methodology, emphasizing stakeholder co-design and complemented by extensive user testing, is an effective and efficient strategy to create a DA for patients living with recurrent diverticulitis facing critical treatment decisions.

## Introduction

Diverticular disease is the eighth most prevalent outpatient gastrointestinal diagnosis with over 2.7 million clinic visits yearly. Diverticulitis accounts for over 216,000 inpatient admissions, with an aggregate cost of US $2.2 billion [1]. While most cases of uncomplicated diverticulitis are treated with antibiotics alone, the incidence of recurrence after an initial episode is as high as 35% [2-7]. A decade ago, elective resection was recommended after 2 episodes of uncomplicated diverticulitis or a single episode in young patients [8]. This practice was based on the idea that recurrence and younger age at onset comprised a more “virulent” syndrome at greater risk for recurrence. These assumptions have recently been challenged and refuted. National guidelines from the American Society of Colon and Rectal Surgeons (ASCRS), in 2020, recommended that “the decision to recommend elective sigmoid colectomy after recovery from uncomplicated acute diverticulitis should be individualized” [9]. However, little guidance is available on how to personalize this decision with each patient.

No evidence indicates the superiority of observation or surgery for recurrent diverticulitis. Surgical complications are well described and include mortality, the need for an ostomy, infection, and other morbidity [10-12]. Conversely, inappropriate observation can lead to continued recurrence with an increased risk of hospitalization, emergency surgery, as well as a decreased quality of life [13,14]. Choosing the option that is most consistent with patient values is critical for optimizing outcomes [15,16]. In addition, there is significant national variation in standardized colon resection ratios for recurrent diverticulitis, with surgeon density and hospital-level factors serving as the main drivers of resection as opposed to patient factors [17]. No research has examined the extent to which shared decision-making is occurring.

Educational decision aids (DAs) and shared decision-making programs have been shown to improve outcomes and reduce decisional conflict in selecting a treatment for diseases, including prostate cancer [18], breast cancer [19], and joint replacement [20]. Recurrent diverticulitis shares several features with joint replacement; both are benign processes where treatment approaches are driven primarily by an improvement in quality of life. Despite this, an extensive literature review of PubMed and Google using the search term “diverticulitis decision aid” identified no current DAs for treatment options for recurrent left-sided diverticulitis. While we hypothesize that such a program would be beneficial for patients with recurrent diverticulitis to improve long-term patient outcomes, function, and satisfaction, there is a foundational gap in the field in that there are no DAs available to support this key aspect of care. The objective of this research is to use rigorous methods, engaging key stakeholders throughout the process, to develop an effective DA to support the selection of either a surgical or monitoring treatment approach in recurrent diverticulitis.

## Methods

From December 12 to 15, 2022, we conducted a 4-day design sprint at Vanderbilt University Medical Center in Nashville, Tennessee (Textbox 1). Adhering to the International Patient Decision Aid Standards (IPDAS) [21,22], we used a modified design sprint methodology developed by Google Ventures (Alphabet Inc) to create a prototype DA for considering colectomy in the setting of diverticular disease [23,24]. Design sprint methodology seeks to condense the potentially months-long production cycle of debate, and instead focus a small team on producing a prototype in just a few days. This approach enabled us to rapidly develop a user-centered solution in the form of a prototype that could be tested and revised iteratively, as done elsewhere [25]. The design sprint team comprised 13 participants from diverse backgrounds, including 4 surgeons, 3 behavioral scientists, 2 physician experts in DA development, 1 expert in health information technology, 4 patients who have undergone medical and surgical treatment of diverticulitis, and 1 caregiver of patients with diverticulitis. Core participants of the design sprint team met in person, and all key points of the design sprint were time-constrained to facilitate the timely completion of the project. KB, a masters-level psychologist with extensive qualitative research experience, facilitated the sprint.

**The 4-day plan for our diverticulitis decision aid design sprint with specific, measurable goals corresponding to each day.**

**Day 1: Defining the problem**

- Identifying goals
- Outlining current understanding and practices
- Selecting sprint target

**Day 2: Ideate**

- Review existing decision aids
- Brainstorming solutions
- Selecting solutions

**Day 3: Storyboard**

- Solution refinement
- Decision aid content refinement

**Day 4: Prototype**

- Development of initial prototype

### Ethical Considerations

Both the design sprint and usability portions of the study were reviewed and approved by the Vanderbilt Institutional Review Board (IRB 220707). Participants involved in the design sprint phase were compensated for their time at a rate of US $20 per hour for patients and US $50 per hour for physicians. Participants involved in the iterative refinement phase were compensated for their time at a rate of US $50 per session for patients and US $200 per session for physicians. This study was not registered as a clinical trial as it only entailed the creation and usability testing of the DA. All data were maintained on a password-encrypted database and the data were analyzed anonymously.

### Decision Aid Design

#### Day 1: Defining the Problem

On day 1, we began by defining the overall goals for our project. Our DA aimed to (1) be a tool for patients to make the best personalized decision possible with the available information; (2) be usable, sustainable, and adaptable; and (3) empower patients and facilitate communication with their surgeons when making a decision regarding colectomy. We then introduced our group’s previous qualitative investigation to assess the key factors that both patients and surgeons evaluate when considering colectomy or observation. The themes identified included limited knowledge about treatment options, difficulty in communication, and uncertainty in important outcomes related to the decision-making process [26,27]. Next, using the input from our core sprint group and stakeholders, we created a “workflow map” outlining the experience of a patient with diverticulitis within the health care system (Multimedia Appendix 1). Drawing upon these themes, we curated a list of potential problems that might arise during each step of the workflow, identifying areas where our DA would be able to improve both patient and clinician decision-making.

We met through videoconference with expert stakeholders (eg, patients, gastroenterologists with a focus on diverticulitis and community surgeons) to assess perceptions of how best to enhance the workflow map and further understand the problems at each node of the patient’s experience within the health care system. Each “problem” was then reframed into an “opportunity” or “question” using the How Might We (HMW) method (Multimedia Appendix 1). The HMW statements were grouped into themes to identify the most useful ideas for building the DA. Major categories identified included (1) facilitating communication, (2) DA characteristics, (3) educational elements, (4) design features, and (5) functionality.

At the end of the conclusion of the first day, we had identified a “sprint target,” a place in the workflow where our group hypothesized the DA would be used. This target was aimed to address the concept: “HMW add efficiency for both the patient and provider?” as the target for the sprint. Thereafter, we frequently revisited our primary goal, reflecting, “Does this design choice meet the need proposed in our goal specifically within the context of our target timeline?”

#### Day 2: Ideate

On day 2, we reviewed existing DA designs from other areas of health care [18,25,28]. These were chosen by our DA expert as examples of high-quality DAs from a range of clinical decisions. They were presented so that the group could get a wide idea of potential elements to include in the diverticulitis DA. We also interviewed sprint group members who had previously designed and implemented DAs to elicit insight into the development process. Next, this information was contextualized in the setting of our goals, and ideation sessions were held in which each member generated as many ideas as possible in the form of sketches or simple drawings to represent the concept (Multimedia Appendix 2). Thereafter, the team voted to select the most promising solutions, using a democratic voting process. A “super voter” was used to break ties, prevent stagnation, and facilitate the creative process. The super voter is an integral part of the design process. To avoid the tendency to move forward with several good ideas, it is the job of the super voter to decide what exactly will be prototyped. The super voter is excluded from the first round of voting for shortlisting of potential options. Once the group has voted and explained their votes, the super voter selects the strongest concepts to move forward. In our instance, the super voter was the principal investigator.

#### Day 3: Storyboard

On day 3, design solutions were critiqued and further refined to better meet the needs of the DA in the context of our patient population (Multimedia Appendix 3). Each solution was analyzed in a group setting, where potential pitfalls and improvements were discussed in turn. At the end of the process, the chosen solutions were adapted into a new workflow of our ideal DA. We reviewed this workflow iteratively until we created a storyboard of both a website and a handout for use by patients.

#### Day 4: Prototype

Finally, on day 4, we created a mock-up of the website and handout. Attention was paid to the content of each webpage, ensuring it would be applicable and understandable to the widest audience possible.

### Usability Study Design

The mockup was further developed by the Vanderbilt design department into a working prototype website and handout. This website underwent review and revision by a group of content and design experts, including members from the original sprint team.

**Figure 1:**
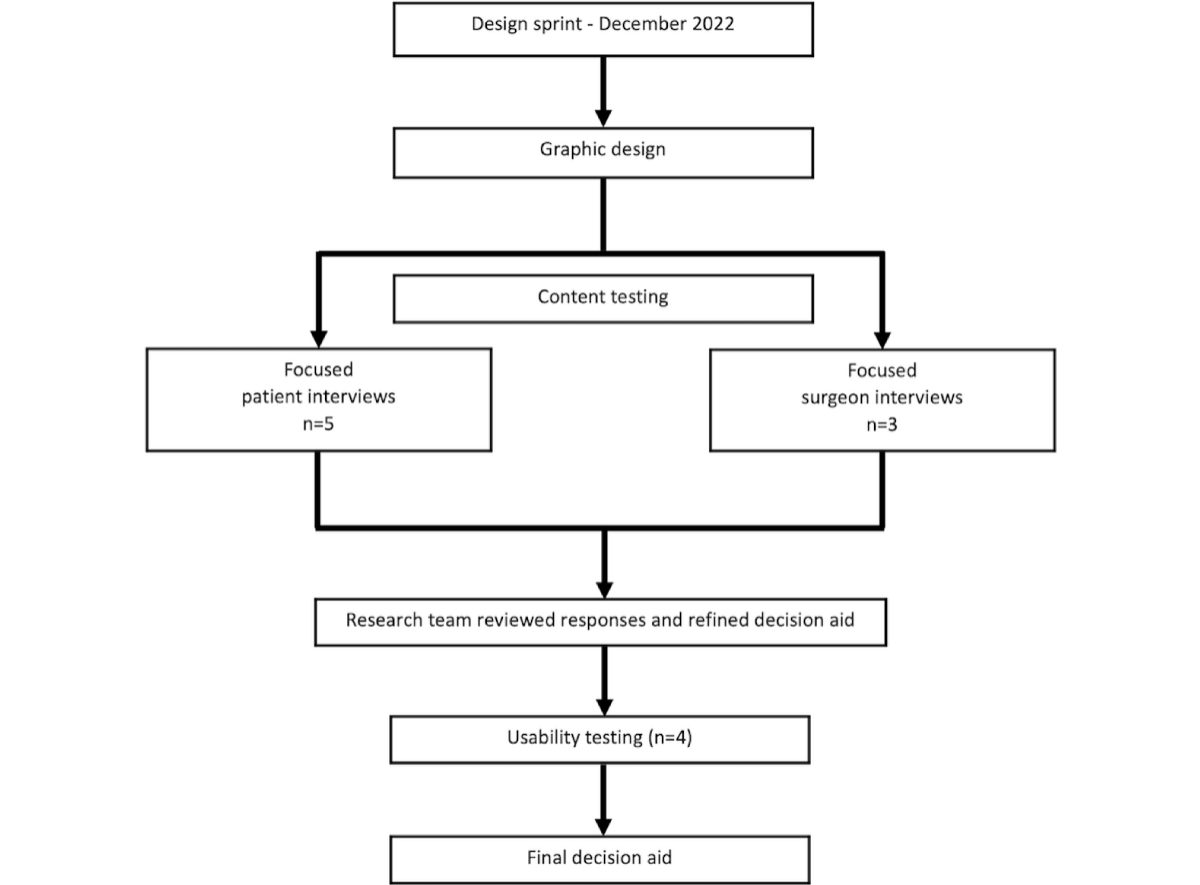
Flowchart demonstrating overall design strategy and steps, beginning with the design sprint and moving toward iterative refinement and usability testing.

We conducted iterative testing and refinement through a mixed methods study using semistructured interviews and surveys from February to May 2023 (Figure 1). Patients were recruited from the Vanderbilt Colorectal Clinic. Inclusion criteria included patients who had previously been seen in consultation for colectomy for recurrent diverticulitis. Exclusion criteria included patients with colo-vesical fistulas, colo-vaginal fistulas, persistent pain, and colonic strictures, as these conditions markedly favor surgical intervention. Semistructured interviews were conducted over videoconferencing using a standardized script developed by a team with experience in qualitative research and patient care (Multimedia Appendix 4). Subjects interacted first with the website and then with the handout. We used video recording to collect responses. We used both scripted questions as well as the think-aloud technique to assess interaction with both elements of the DA. Patients also completed surveys, including the Net Promoter Score (1-item) [29], the System Usability Scale (SUS; 10-item) [30], and the Cultural Sensitivity Questionnaire (10-item) [31].

### Analysis Plan

General satisfaction scores were analyzed using top box scoring with the 2 top scores on a 7-point Likert scale considered to be “top box.” The composite SUS score was calculated by reverse scoring even-numbered items so that all items were scored in the same direction. Composite scores were calculated by summing the item responses and multiplying them by 2.5 so that they fell on a scale of 0-100, with 100 representing the greatest usability. Summary statistics were calculated for the composite scores, and frequencies were reported for the overall rating of user-friendliness. The Cultural Sensitivity Questionnaire was calculated by dividing the score for each question by the adjusted question number. A category score greater than 2.5 denotes an acceptable category, and scores of 2.5 or less denote unacceptable categories.

For analysis of the open-ended interview questions, the responses were organized by group (eg, barriers and facilitators) and a thematic analysis of all responses was conducted based on the steps outlined by Braun and Clarke [32]. First, we reviewed all the responses and generated initial themes and categories. These categories were reviewed by 2 of the authors (AF and KB) and confirmed by a third author (AH). Finally, we categorized the themes to provide a description and examples in this report. We quantified the comments in each category to provide a frequency related to participants’ ideas, suggestions, and ideas related to the usability of the DA.

After revisions from this group, the DA underwent iterative usability testing using focused interviews with both patients and surgeons of the target population. In addition to the sprint participants, we met with experts and stakeholders outside the group, including a community surgeon, health care professionals with experience in designing and implementing DAs, and additional patients and caregivers.

## Results

At the end of the design sprint, we successfully developed a prototype of a diverticulitis DA in the form of a complimentary interactive website and handout. The DA has 3 discrete sections.

**Figure 2:**
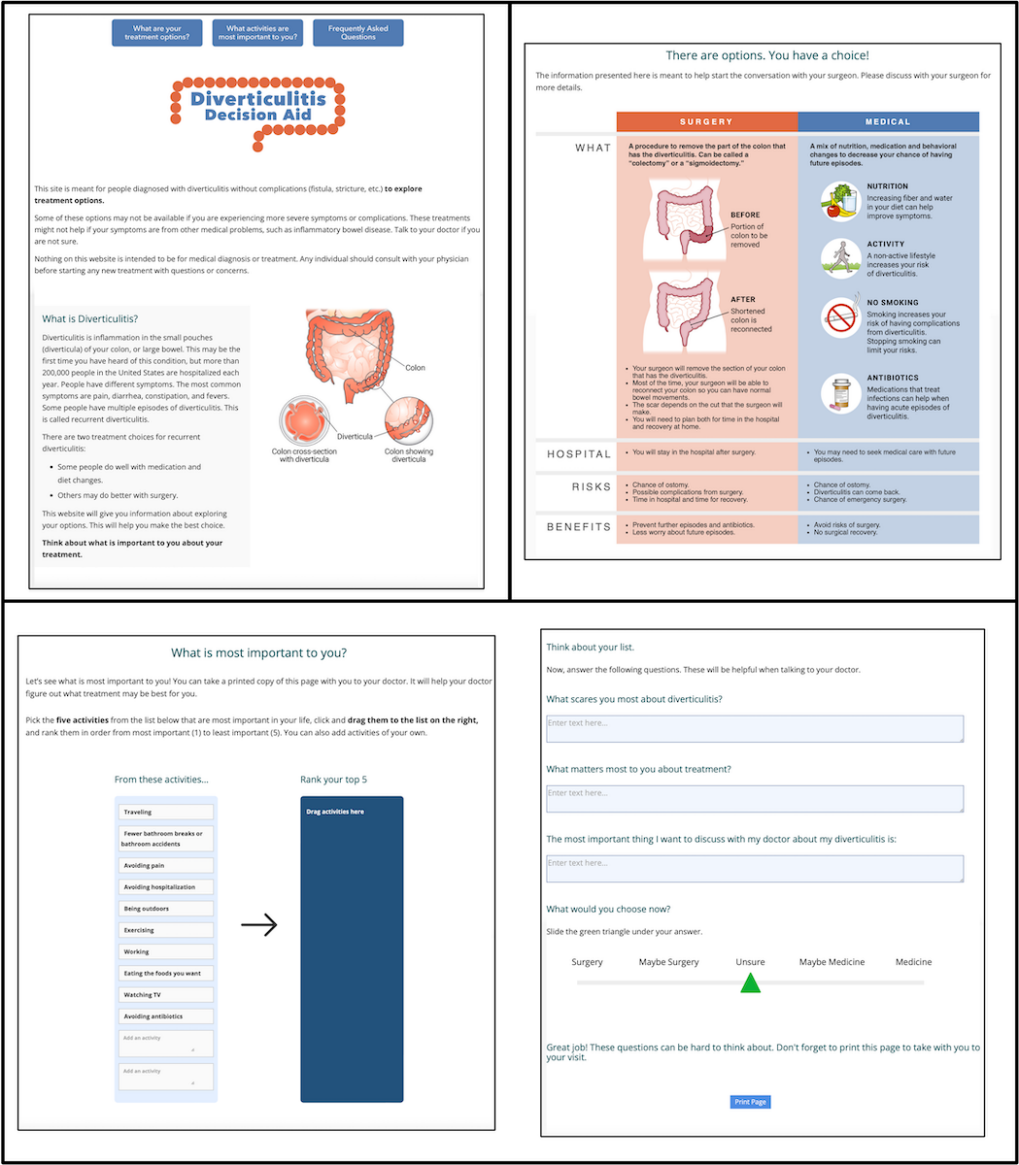
Graphic depicting the diverticulitis decision aid website pages. The first section contains background information about diverticulitis. The second section explains the different treatment options. The third section is a values clarification exercise for patients.

The first section (Figure 2) includes background information designed to increase patient’s knowledge about diverticulitis as a disease entity and to introduce the treatment options of colectomy and observation. This section provides a generic working definition of diverticulitis as a disease process and encourages patients to engage with the website while reflecting on their values regarding their treatment.

The second section (Figure 2) includes detailed information about each treatment option and a section on the risks and benefits of surgery and observation. This section provides patients with a framework for what their life might look like after colectomy or observation, empowering them with information to reflect on before their surgical appointment. After patients gain a general understanding of the disease process and their options, they proceed to the interactive portion of the DA.

In the third section (Figure 2), patients are invited to engage in a values clarification exercise that will help them prepare for a discussion with their surgeon. They are instructed to pick the 5 activities that are most meaningful in their lives and rank them in order from most to least important. Thereafter, patients are provided the opportunity to engage in self-reflection regarding fears, priorities, and important topics they want to discuss with their surgeon concerning their diverticular disease, through several open-ended questions to document their answers before their appointment. Finally, on a Likert scale between “surgery,” “maybe surgery,” “unsure,” “maybe medicine,” and “medicine,” patients are asked to pick what they would choose now for their treatment option after reading the relevant background information and completing the DA. Following completion, patients can print as well as email a report of this activity.

Patients are expected to bring their completed clarification exercise to their clinic appointment to help initiate the conversation with their surgeon. The DA serves as a guide for shared decision-making and equips surgeons with the relevant information to help guide patients in seeking the best individualized treatment for their recurrent uncomplicated diverticulitis.

After initial drafting based on the design sprint prototype, the diverticulitis DA underwent 2 phases of usability testing with patients. First, semistructured interviews were conducted with both patients and surgeons with associated surveys. Table 1 reports patient demographics for both rounds (Table 1). Table 2 reports patient satisfaction with the DA from the initial round (Table 2). Overall, patients reported being highly satisfied with both the website and the handout. Regarding the Net Promoter Scale, 80% reported that they would be “extremely likely to recommend website”. The SUS scores were both >90%. The Cultural Sensitivity Questionnaire mean score was 3.7 (SD 0.16), indicating acceptable sensitivity. Almost universally, surgeons were positive about the tool and in agreement with the content and material. Illustrative quotes from the patient and surgeon interviews are reported in Tables 3 and 4, respectively.

**Table 1:** Baseline demographics of patients who participated in both rounds of usability testing of the diverticulitis decision aid prototype.

Variables			Total (n=9)		Round 1 (n=5)		Round 2 (n=4)
Age (years), mean (SD)			56.7 (8.6)		58.2 (10.2)		55 (6)
**Gender, n (%)**							
	Female	4 (44)		2 (40)		2 (50)	
**Race, n (%)**							
	White	6 (67)		3 (60)		3 (75)	
	African American	3 (33)		2 (40)		1 (25)	
**Education, n (%)**							
	College graduate	6 (67)		3 (60)		3 (75)	
	Postgraduate degree	3 (33)		2 (40)		1 (25)	
**Employment status, n (%)**							
	Full time	7 (78)		4 (80)		3 (75)	
	Retired	2 (22)		1 (20)		1 (25)	
**Insurance, n (%)**							
	Private	6 (67)		4 (80)		2 (50)	
	Other	3 (33)		1 (20)		2 (50)	
**Health literacy support, n (%)**							
	Never	9 (100)		5 (100)		4 (100)	
**Health status, n (%)**							
	Good	2 (22)		1 (20)		1 (25)	
	Very good	5 (56)		3 (60)		2 (75)	
	Excellent	2 (22)		1 (20)		1 (25)	

**Table 2:** Patient survey responses after the initial round of usability testing of the diverticulitis decision aid.

Survey responses			Values
**Satisfaction scale,** **n (%)**			
	Overall, I am satisfied with the ease of completing the task in this scenario, top box	5 (100)	
	Overall, I am satisfied with the amount of time it took to complete the task in this scenario, top box	5 (100)	
	Overall, I am satisfied with the support information when completing this task, top box	4 (80)	
	Rate your overall experience with this website, top box	5 (100)	
	Rate your overall experience with this handout, top box	5 (100)	
**Net Promoter Scale, n (%)**			
	Extremely likely to recommend the website	4 (80)	
**System Usability Scale, mean (SD)**			
	Website	93 (4.1)	
	Handout	93 (4.1)	
Cultural Sensitivity Questionnaire, mean (SD)			3.7 (0.16)

**Table 3:** Illustrative quotes from patient-focused interviews during content testing of the diverticulitis decision aid.

Themes	Web page					Handout
	Home page	Options	Value clarifications	FAQ^a^		
Initial impression	“Very much about the patient, empowers the patient”	“I am a number/details guy and I would like a little more detail.”	“Gives you talking points when going into your doctor’s visit.”	“Very helpful; I like FAQ and Q&As to give an introduction.”	“I could fill this out in a doctor’s office.”	
Content	“I didn’t realize there was more below it”	“I like the images. Simple but shows exactly what it is.”	“Pain was the primary symptom, not on the list.”	“Can you write from the patient’s point of view?”	“Is this really any different than the website? If I had this, would not use the website.”	
Navigation	“Navigates immediately to options, does not scroll down to view home page”	“Navigates to next page using bottom button without issue.”	“Easy to navigate back to from other pages.”	“Big red “forward/backward” arrow might be good for navigation.”	“The handout does not tell you there is a backside.”	

^a^FAQ: frequently asked question.

**Table 4:** Illustrative quotes from surgeon-focused interviews during content testing of the diverticulitis decision aid.

Themes	Web page					Handout
	Home page	Options	Value clarifications	FAQ^a^		
Initial impression	—^b^	“Overall good overview.”	“Meant to get at the root of the problem.”	—	—	
Content	“Simple and straightforward.”	“Chance of Ostomy” as first option is kind of fear-mongering as it is very unlikely.	“Did not initially understand click and drag.”	“Like the simplicity, do not want it to be too complicated or onerous for the patient.”	“Would work best if they had it before surgical consultation. If they could fill it out either at home or in the waiting room waiting to see the surgeon.”	
Navigation	“Proceeds to treatment options immediately.”	“Navigates easily to next page using bottom button.”	“Navigates to FAQ.”	“Navigates back to previous pages using the top bar easily.”	—	

^a^FAQ: frequently asked question.

^b^Not applicable.

## Discussion

We applied a modified version of the design sprint methodology to create an educational decision aid in the form of a deliverable webpage and handout aimed at facilitating communication between patients and surgeons alike. By using this design approach, we rapidly created a prototype that is constructed to be informative, sustainable, adaptable, and widely applicable. Our previous research identified several key decisional needs in the complex decision-making process that we made sure to address in our final product [26,27]. We incorporated feedback from our expert consultants, stakeholders, and patients to ensure the decision aid was informative yet easy to use and relevant. Examples of this include changing the website navigation to make it more intuitive, the addition of pain to the values clarification exercise, and additional content regarding ostomies to both the information portion and the FAQ sections. Assessments of satisfaction and usability indicated high levels of both with adequate cultural sensitivity.

This study has several strengths. This is the first DA designed to facilitate difficult conversations between surgeons and patients with recurrent uncomplicated diverticulitis about treatment options per American Society of Colon and Rectal Surgeons guidelines [9]. Our design process offers a road map for quality DA development that builds on International Patient Decision Aid Standards guidelines, incorporates feedback from stakeholders, and highlights the importance of iterative usability testing. Initial testing indicates high levels of satisfaction and usability. In addition, the DA is online, which makes it relatively easy to update as information changes. This allows for easier integration with health care systems and dissemination to patients before a visit.

There are limitations to this study. The initial design sprint was performed at a single academic medical center, which raises concerns about generalizability. Although a standard 5-day approach is recommended as initially developed by Google Ventures [23,24], this relies on the premise that participants have a strong understanding of the disease process as well as user needs and challenges. Additional time or informative sessions may be needed to lay the necessary groundwork before initiating the sprint design process.

While we present the initial development of this DA here, additional work is required before it may be integrated into clinical practice. The next steps include feasibility testing and preliminary data collection on the implementation of the DA in the form of a pilot trial, followed by a multicenter, randomized controlled trial comparing the decision aid with standard clinical consultation. During these stages, we will use focused interviews of both providers and patients to further improve the tool. Should efficacy be demonstrated, an implementation study will be crucial to ensure appropriate integration into clinical workflow.

Our study illustrates the use of a modified version of a design sprint methodology to create a DA for patients with recurrent diverticulitis considering colectomy aimed at facilitating communication between patient and clinician that was well received by patients. Our experience with this method illustrates the value of the design sprint methodology in the creation of tools to improve the overall care of patients.

## Appendix

Multimedia Appendix 1 Day 1 images.

Multimedia Appendix 2 Day 2 images – ideate with voting.

Multimedia Appendix 3 Day 3 image - storyboarding.

Multimedia Appendix 4 Usability testing interview script.

## Abbreviations

ASCRS: American Society of Colon and Rectal Surgeons
DA: decision aid
HMW: How Might We
IPDAS: International Patient Decision Aid Standards
IRB: institutional review board
SUS: System Usability Scale

## Data Availability

The data sets generated during and/or analyzed during this study are available from the corresponding author on reasonable request.

## References

[1] Peery AF, Crockett SD, Murphy CC, Lund JL, Dellon ES, Williams JL, Jensen ET, Shaheen NJ, Barritt AS, Lieber SR, Kochar B, Barnes EL, Fan YC, Pate V, Galanko J, Baron TH, Sandler RS (2019). Burden and Cost of Gastrointestinal, Liver, and Pancreatic Diseases in the United States: Update 2018. Gastroenterology.

[2] Ambrosetti P (2012). Value of CT for acute left-colonic diverticulitis: the surgeon's view. Dig Dis.

[3] Anaya DA, Flum DR (2005). Risk of emergency colectomy and colostomy in patients with diverticular disease. Arch Surg.

[4] Broderick-Villa G, Burchette RJ, Collins JC, Abbas MA, Haigh PI (2005). Hospitalization for acute diverticulitis does not mandate routine elective colectomy. Arch Surg.

[5] Eglinton T, Nguyen T, Raniga S, Dixon L, Dobbs B, Frizelle FA (2010). Patterns of recurrence in patients with acute diverticulitis. Br J Surg.

[6] Hall JF, Roberts PL, Ricciardi R, Read T, Scheirey C, Wald C, Marcello PW, Schoetz DJ (2011). Long-term follow-up after an initial episode of diverticulitis: what are the predictors of recurrence?. Dis Colon Rectum.

[7] Shaikh S, Krukowski ZH (2007). Outcome of a conservative policy for managing acute sigmoid diverticulitis. Br J Surg.

[8] Roberts P, Abel M, Rosen L, Cirocco W, Fleshman J, Leff E, Levien D, Pritchard T, Wexner S, Hicks T (1995). Practice parameters for sigmoid diverticulitis. The Standards Task Force American Society of Colon and Rectal Surgeons. Dis Colon Rectum.

[9] Hall J, Hardiman K, Lee S, Lightner A, Stocchi L, Paquette IM, Steele SR, Feingold DL, Prepared on behalf of the Clinical Practice Guidelines Committee of the American Society of ColonRectal Surgeons (2020). The American Society of Colon and Rectal Surgeons Clinical Practice Guidelines for the Treatment of Left-Sided Colonic Diverticulitis. Dis Colon Rectum.

[10] Collins D, Winter DC (2008). Elective resection for diverticular disease: an evidence-based review. World J Surg.

[11] Pessaux P, Muscari F, Ouellet J, Msika S, Hay J, Millat B, Fingerhut A, Flamant Y (2004). Risk factors for mortality and morbidity after elective sigmoid resection for diverticulitis: prospective multicenter multivariate analysis of 582 patients. World J Surg.

[12] Simianu VV, Flum DR (2014). Rethinking elective colectomy for diverticulitis: a strategic approach to population health. World J Gastroenterol.

[13] Buchs NC, Konrad-Mugnier B, Jannot A, Poletti P, Ambrosetti P, Gervaz P (2013). Assessment of recurrence and complications following uncomplicated diverticulitis. Br J Surg.

[14] Stollman N, Raskin JB (2004). Diverticular disease of the colon. Lancet.

[15] Forgione A, Leroy J, Cahill RA, Bailey C, Simone M, Mutter D, Marescaux J (2009). Prospective evaluation of functional outcome after laparoscopic sigmoid colectomy. Ann Surg.

[16] van de Wall BJM, Stam MAW, Draaisma WA, Stellato R, Bemelman WA, Boermeester MA, Broeders IAMJ, Belgers EJ, Toorenvliet BR, Prins HA, Consten ECJ, DIRECT trial collaborators (2017). Surgery versus conservative management for recurrent and ongoing left-sided diverticulitis (DIRECT trial): an open-label, multicentre, randomised controlled trial. Lancet Gastroenterol Hepatol.

[17] Hawkins AT, Samuels LR, Rothman RL, Geiger TM, Penson DF, Resnick MJ (2022). National Variation in Elective Colon Resection for Diverticular Disease. Ann Surg.

[18] Ankolekar A, Vanneste BGL, Bloemen-van Gurp E, van Roermund JG, van Limbergen EJ, van de Beek K, Marcelissen T, Zambon V, Oelke M, Dekker A, Roumen C, Lambin P, Berlanga A, Fijten R (2019). Development and validation of a patient decision aid for prostate Cancer therapy: from paternalistic towards participative shared decision making. BMC Med Inform Decis Mak.

[19] Whelan T, Levine M, Willan A, Gafni A, Sanders K, Mirsky D, Chambers S, O'Brien MA, Reid S, Dubois S (2004). Effect of a decision aid on knowledge and treatment decision making for breast cancer surgery: a randomized trial. JAMA.

[20] Sepucha K, Bedair H, Yu L, Dorrwachter JM, Dwyer M, Talmo CT, Vo H, Freiberg AA (2019). Decision Support Strategies for Hip and Knee Osteoarthritis: Less Is More: A Randomized Comparative Effectiveness Trial (DECIDE-OA Study). J Bone Joint Surg Am.

[21] Coulter A, Stilwell D, Kryworuchko J, Mullen PD, Ng CJ, van der Weijden T (2013). A systematic development process for patient decision aids. BMC Med Inform Decis Mak.

[22] Elwyn G, O'Connor A, Stacey D, Volk R, Edwards A, Coulter A, Thomson R, Barratt A, Barry M, Bernstein S, Butow P, Clarke A, Entwistle V, Feldman-Stewart D, Holmes-Rovner M, Llewellyn-Thomas H, Moumjid N, Mulley A, Ruland C, Sepucha K, Sykes A, Whelan T, International Patient Decision Aids Standards (IPDAS) Collaboration (2006). Developing a quality criteria framework for patient decision aids: online international Delphi consensus process. BMJ.

[23] Banfield RL, Wax T (2015). Design Sprint: A Practical Guidebook for Building Great Digital Products.

[24] Knapp JZ, Kowitz B (2016). Sprint: How to Solve Big Problems and Test New Ideas in Just Five Days.

[25] Martinez W, Threatt AL, Rosenbloom ST, Wallston KA, Hickson GB, Elasy TA (2018). A Patient-Facing Diabetes Dashboard Embedded in a Patient Web Portal: Design Sprint and Usability Testing. JMIR Hum Factors.

[26] Hawkins AT, Penson DF, Geiger TM, Bonnet KR, Mutch MG, Maguire LH, Schlundt DG, Rothman RL (2024). The Patient Perspective on Colectomy for Recurrent Diverticulitis: A Qualitative Study. Ann Surg.

[27] Hawkins AT, Rothman R, Geiger TM, Bonnet KR, Mutch MG, Regenbogen SE, Schlundt DG, Penson DF (2022). Surgeons' Perspective of Decision Making in Recurrent Diverticulitis: A Qualitative Analysis. Ann Surg Open.

[28] Tate CE, Venechuk G, Pierce K, Khazanie P, Ingle MP, Morris MA, Allen LA, Matlock DD (2021). Development of a Decision Aid for Patients and Families Considering Hospice. J Palliat Med.

[29] Brown MI (2020). Comparing the validity of net promoter and benchmark scoring to other commonly used employee engagement metrics. Human Resource Dev Quarterly.

[30] Brooke J (1996). Usability Evaluation in Industry.

[31] Guidry JJ, Walker VD (1999). Assessing cultural sensitivity in printed cancer materials. Cancer Pract.

[32] Braun V, Clarke V (2006). Using thematic analysis in psychology. Qualitative Research in Psychology.

