# Supplementary figures and images for: Decision Aid for Colectomy in Recurrent Diverticulitis: Development and Usability Study
Source: JMIR Form Res. 2024 Sep 3;8:e59952. doi: 10.2196/59952 (PMC11408895; doi:10.2196/59952)

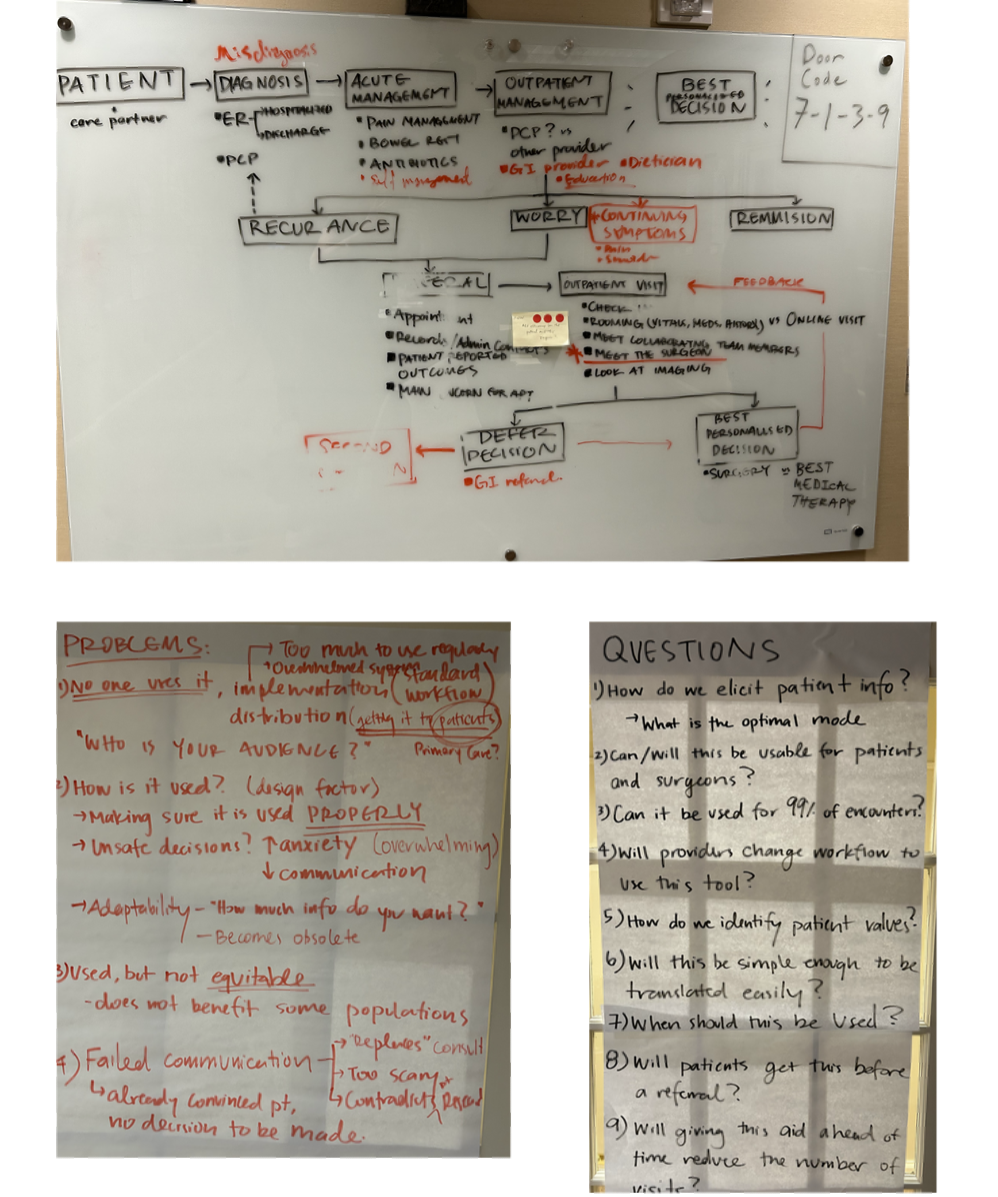

Supplement: Multimedia Appendix 1 [file formative_v8i1e59952_app1.png]

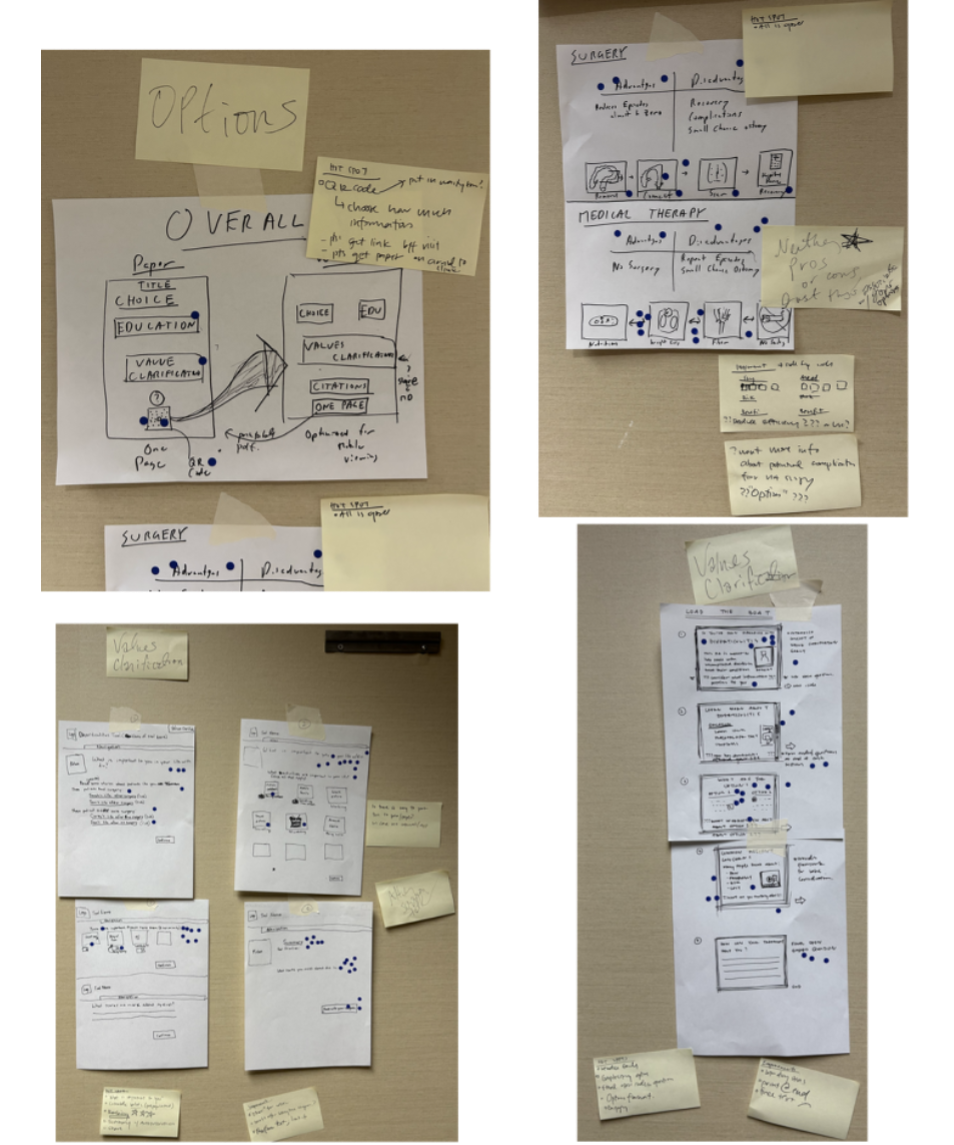

Supplement: Multimedia Appendix 2 [file formative_v8i1e59952_app2.png]

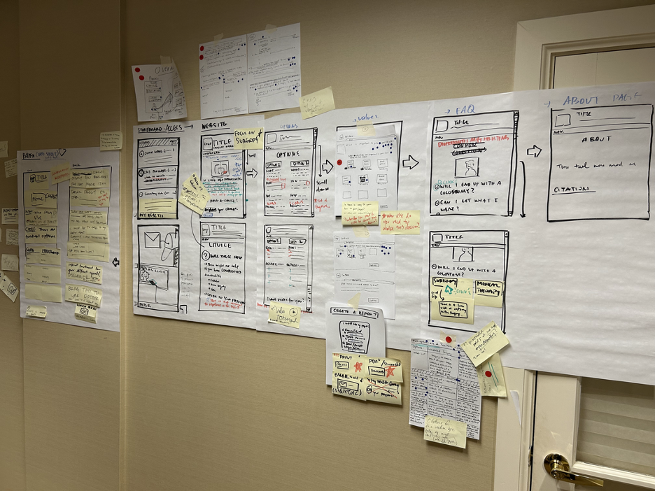

Supplement: Multimedia Appendix 3 [file formative_v8i1e59952_app3.png]
