# Supplementary material for: Decision Aid for Colectomy in Recurrent Diverticulitis: Development and Usability Study
Source: JMIR Form Res. 2024 Sep 3;8:e59952. doi: 10.2196/59952 (PMC11408895; doi:10.2196/59952)
Supplement: Multimedia Appendix 4 [file formative_v8i1e59952_app4.docx]

**WEBSITE**

Interview Instructions

- Now we will move on to the website
- We will start by having you take a few minutes to get familiar with the website
- Please talk out loud as you’re looking at this website and moving through it. Feel free to share your initial thought process and impressions; there are no right or wrong answers.
- Then I’ll ask you a few questions about your experience both overall and more specifically
- Please take some time to review this website

[Ask for each website page]

Initial Impressions

- Overall, what is your first impression of this page?
- What is this page meant to do?

Navigation

- What made this page hard to navigate?
- What would make it easier to navigate?

Content

- What do you think of the content of this page?
- What topics do you think were the most helpful?
- What content is hard to understand?
- What content is missing?
- What content should we cut?

**Searching Tasks**

1. What are the pros/cons of surgery for diverticulitis?
2. What are some things you need to consider when thinking about surgery/medical treatment? (values clarification page)
3. What can you eat if you have diverticulitis?

 If participant lands on wrong page, ask (as needed):

- Did this page answer that question?
- Can you tell me where on this page the answer is?
- Is there anywhere else you can look that might answer the question better?

When participant lands on correct page ask:

- As you navigated your way to [the correct page], what navigation steps should have happened but didn’t?
- What navigation steps did happen, but shouldn’t have?

For each question, prompt the following response.


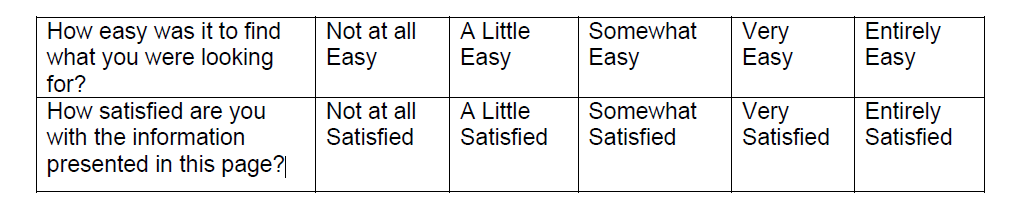
**Overall**

- How useful was this website for people with diverticulitis?
- How can we make this website more user-friendly to people navigating the website?
- What can we improve in the content?
- What section was most helpful?
- What section was least helpful?
- How do you see yourself using this website?

**After scenario questionnaire🡪 REDCap?**

For the next three questions please rate how much you agree or disagree with each statement that I will read, rating from 1 (strongly agree) through 7 (strongly disagree).

1. Overall, I am satisfied with the ease of completing the task in this scenario.

2. Overall, I am satisfied with the amount of time it took to complete the task in this scenario.

3. Overall, I am satisfied with the support information (on-line help, messages, documentation) when completing this task. [Re: #3, If participant asks: “Support information” can mean the glossary, the references/reference citations, etc.]

**Satisfaction score** (1-5)

How satisfied are you with your experience with this website?


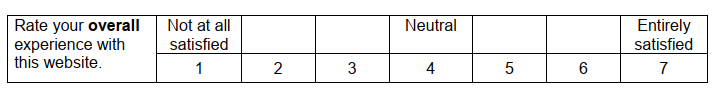


**Net promotor Score** (NPS)


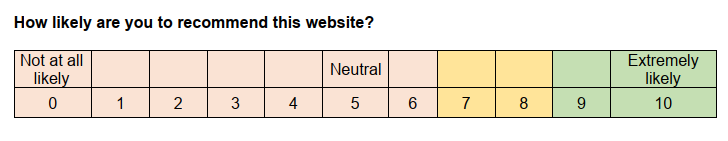
**SUS Score (usability)**


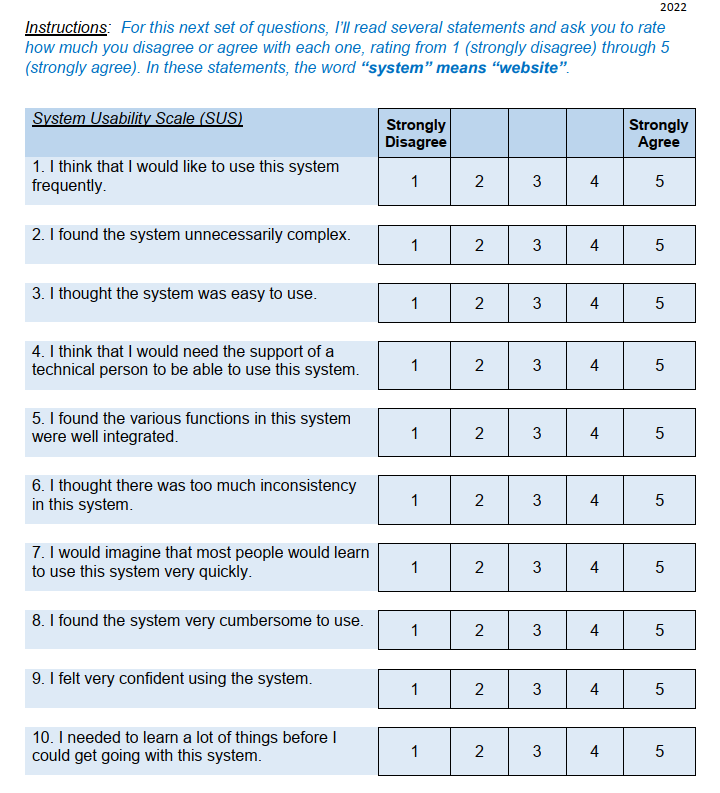
**HANDOUT**

Interview Instructions

- We will start by having you take a few minutes to get familiar with [the handout].
- Then I’ll ask you a few questions about your experience both overall and more specifically
- Think out loud
- Please take some time to review this handout

Initial Impressions

- Overall, what is your first impression of this handout?
- Can you describe to me what the purpose of this handout is?

Readability

- What do you think about the organization of the handout?
- What is confusing in the instructions?

Content

- What do you think of the content of this handout?
  - - What content is hard to understand?
    - What content is missing?
    - What content should we cut?
- What topics do you think were the most helpful?

**Overall**

1. What were your expectations for this handout?
   1. How useful is this handout with regards to the website?
2. How could you see yourself using this handout?
3. What suggestions do you have for improving handout content, navigation, and design?

5. What section of the handout interested you most?

6. What section do you think people will be most drawn to?

REDCAP [do not go to the next section until prompted by the interviewer]

How satisfied are you with your experience with this handout?


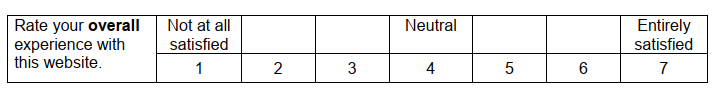
**Net Promoter Score (NPS)**

How likely are you to recommend this handout?

| Not at all likely |  |  |  |  | Neutral |  |  |  |  | Extremely likely |
| --- | --- | --- | --- | --- | --- | --- | --- | --- | --- | --- |
| 0 | 1 | 2 | 3 | 4 | 5 | 6 | 7 | 8 | 9 | 10 |

ENDING QUESTIONS:

Cultural Sensitivity

For the next set of questions, I will ask how much you agree or disagree with each statement I read. The response options are: Strongly Agree, Agree, Neutral/Unsure, Disagree, and Strongly Disagree.


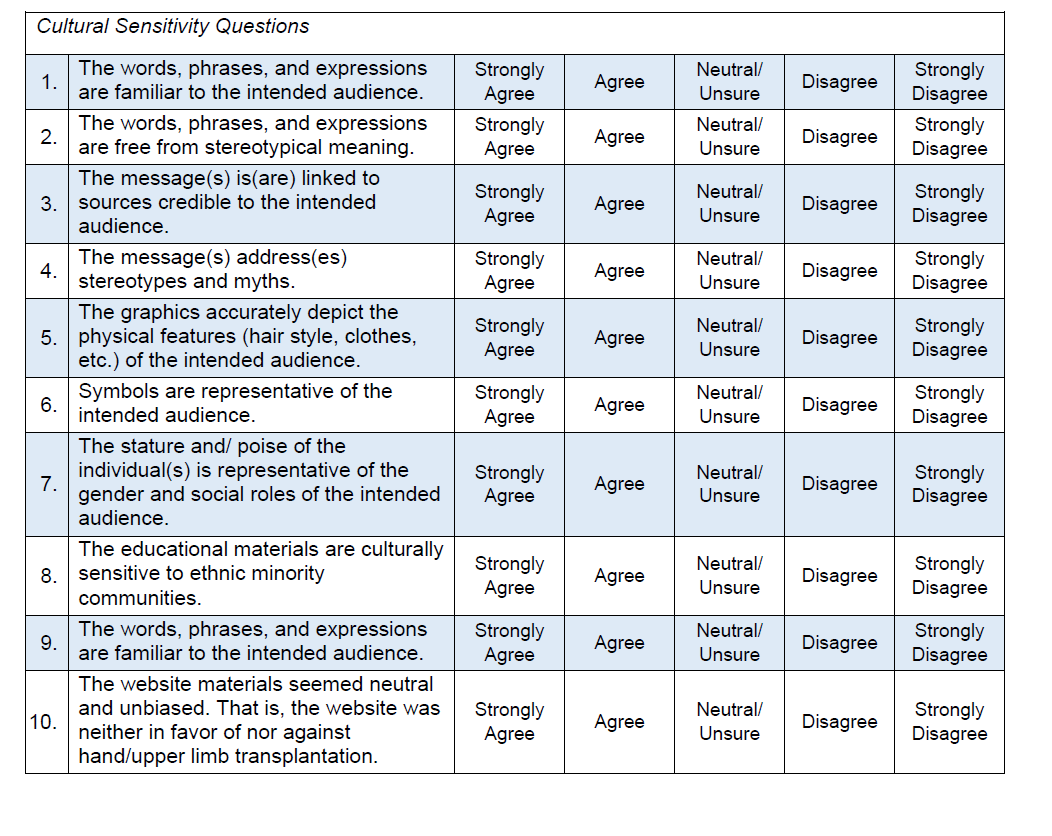
What can we do to improve the cultural sensitivity of the website or handout?

**Demographics: (at end)**

- Age
- Gender
- Hispanic/Latino
- Race
- Marital Status
- Education
- Employment status
- Occupation
- Income
- Health Insurance
- Do you have someone to help you when reading instructions/processing information?
- Health Status
- First episode of diverticulitis
- How often do you have diverticulitis episodes?
- Did you have surgery? When, what. Experience with ostomy
- Questions/Computations
